# The causality between gut microbiome and chronic regional pain: a Mendelian randomization analysis

**DOI:** 10.3389/fmicb.2024.1329521

**Published:** 2024-02-29

**Authors:** Qing-Ao Xiao, Lei Qin, Jing Yu, Yin-Tao Hu, Lin-Feng Ai, De-Cheng Wang, Xuan Xia, Xiao-Lin Zhang

**Affiliations:** ^1^Department of Interventional Radiology, The First College of Clinical Medical Science, China Three Gorges University, Yichang, Hubei, China; ^2^Department of Interventional Radiology, Yichang Central People’s Hospital, Yichang, Hubei, China; ^3^Hubei Key Laboratory of Tumor Microenvironment and Immunotherapy, College of Basic Medical Sciences, China Three Gorges University, Yichang, China; ^4^Institute of Infection and Inflammation, China Three Gorges University, Yichang, Hubei, China; ^5^Department of Physiology and Pathophysiology, College of Basic Medical Science, China Three Gorges University, Yichang, Hubei, China

**Keywords:** gut microbiome, Mendelian randomization, chronic regional pain, causality, genome-wide association study

## Abstract

**Background:**

Numerous investigations have underscored the causal effect between chronic pain (CP) and gut microbiota, jointly contributing to the onset and development of widespread CP. Nonetheless, there was still uncertainty about the causal effect between gut microbiota and chronic regional pain (CRP).

**Methods:**

Genome-wide association study (GWAS) summary data of gut microbial taxa (MiBioGen Consortium: 211 microbiotas and the Dutch Microbiome Project: 207 microbiotas) and eight types of CRP were used to reveal the causal effect between persistent pain in a specific region of the body and gut microbiota. A two-sample bidirectional Mendelian randomization (MR) design was used. In order to ensure the accuracy of the results, multiple sensitivity analyses were employed.

**Results:**

This study uncovered significant causal associations between six gut microbial taxa and three types of CRP (forward: *Genus Parabacteroides* for general pain; *Class Bacteroidia*, *Order Bacteroidales,* and *Phylum Bacteroidetes* for back pain. Reverse: knee pain for *Genus Howardella* and *Order Coriobacteriales*) by forward and reverse MR analysis. These findings had been verified by a rigorous Bonferroni correction. Furthermore, this research identified 19 microbial taxa that exhibited potential correlations with four types of CRP. There are no significant or potential gut microbiotas that were associated with other types of CRP, including fascial pain, stomach or abdominal pain, and hip pain.

**Conclusion:**

This two-sample bidirectional MR analysis unveiled the causality between gut microbial taxa and eight CRP conditions. The findings reveal the interplay between CRP and 6 gut microbiotas while also delineating 19 potential specific microbial taxa corresponding to diverse locations of persistent pain.

## Graphical Abstract

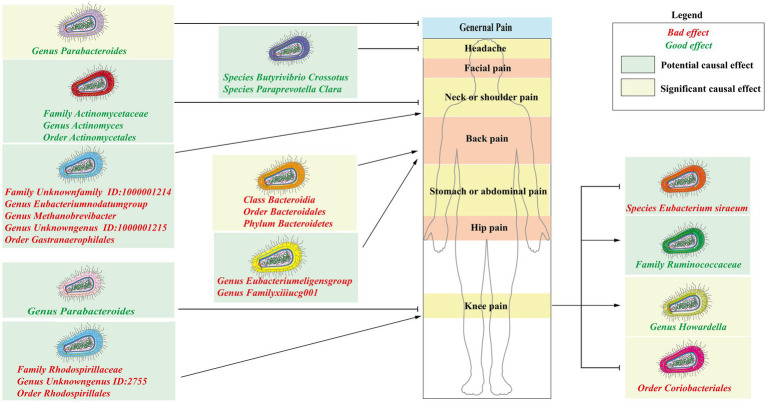

## Highlights

- Gut microbes play a causal role in chronic regional pain (CRP).
- Six gut microbes had a significant impact on three subtypes of CRP, including general pain, back pain, and knee pain.
- Nineteen microbial taxa are potentially associated with four subtypes of CRP, including headache, neck or shoulder pain, back pain, and knee pain.

## Introduction

Chronic pain (CP), according to the definition of the International Association for the Study of Pain (IASP), is defined as persistent pain lasting for more than 3 months (Cohen et al., 2021). CP encompasses an unfavorable sensory and emotional encounter linked with, or similar to, real or potential tissue harm (Cohen et al., 2021). CP emerges as a clinical dilemma on the global horizon. Contemporary research underscores that over 30% of the global population is affected by CP (Cohen et al., 2021). However, until now, effective therapeutic approaches and interventional differentiation for CP remain lacking (Liu et al., 2023).

In the past decade, studies have progressively shed light on the crucial role of gut dysbiosis in the origin and advancement of various diseases (Tilg et al., 2016). Emerging evidence hints at the potential and pivotal role of the gut microbiota in distinct subtypes of CP, including headaches (Chen et al., 2019), abdominal pain (Newlove-Delgado et al., 2019), chronic widespread musculoskeletal pain (Freidin et al., 2021), and neuropathic discomfort (Yang et al., 2019). The research suggests that a dysregulation in the balance between beneficial and harmful bacteria in the gut can lead to an increase in intestinal permeability, allowing inflammatory substances and immune antigens to enter the body. This, in turn, results in the occurrence of systemic inflammation (Di Vincenzo et al., 2023). Such persistent and low-intensity chronic inflammation is considered a significant factor in the development of CP (Zhou et al., 2021). Nevertheless, currently, there is a lack of comprehensive causal association analysis between gut microbiota and chronic regional pain (CRP), such as neck pain and shoulder pain. In addition, ongoing research is susceptible to diverse confounding elements, spanning factors such as nutritional status, sleep cycles, and physical activity (Gonzalez-Alvarez et al., 2023). The intricate associations between distinct gut microbiota compositions and CP further complicate the exploration of their interplay. Based on the above background, we propose a hypothesis: there is an association between gut microbiota and distinct subtypes of CRP.

**Figure 1:**
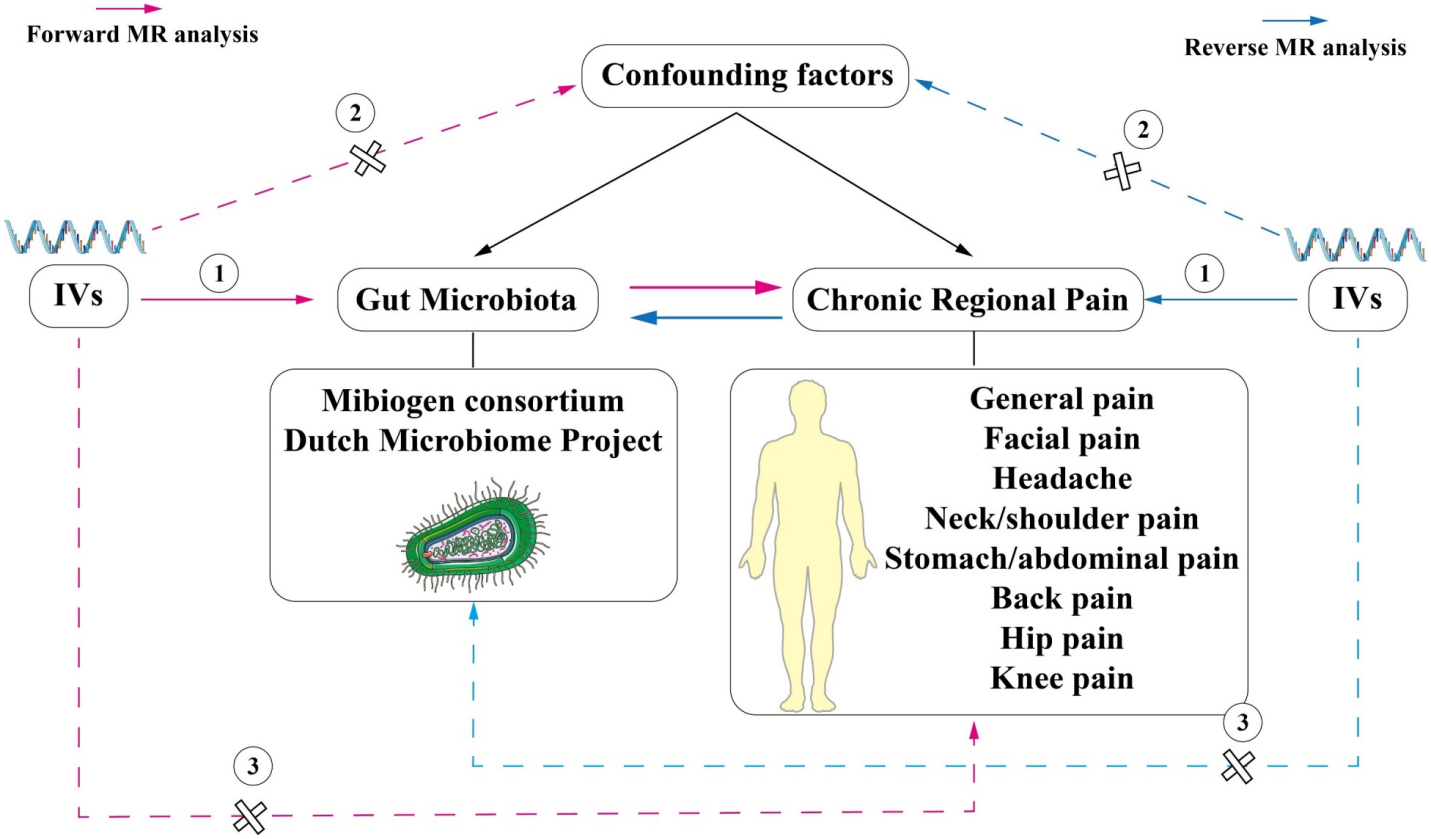
Schematic representation of the two-sample bi-directional Mendelian randomization analysis In this study, single nucleotide polymorphisms (SNPs) strongly associated with exposure were used as instrumental variables (IVs) to explore the causal association between CRP and the gut microbiome. MR analysis was performed under three basic assumptions: (1) IVs must be strongly correlated with exposure; (2) IVs cannot be correlated with confounding factors; (3) IVs can only affect outcomes through exposure factors. The pink lines represent forward MR analysis, while the blue lines represent reverse MR analysis.

Mendelian randomization (MR) is a novel method of genetic epidemiology. It used instrumental variables (IVs), which are strongly associated with exposure, and IVs to make causal inferences and excluded confounding factors (Emdin et al., 2017; Davies et al., 2018). Utilizing MR to investigate causality necessitates satisfying three fundamental assumptions. First, IVs must exhibit a robust correlation with the exposure; second, IVs should not be correlated with confounding factors; and third, IVs can only impact outcomes through exposure (Figure 1; Bowden and Holmes, 2019). Furthermore, single-nucleotide polymorphisms (SNPs) could influence phenotypes, and the inability of phenotypes to alter SNPs allowed MR to eliminate interference from reverse causation (Bowden et al., 2015). In its succinct essence, MR can efficiently alleviate the influence of confounding and reverse causation (Emdin et al., 2017; Davies et al., 2018).

In conclusion, our study systematically investigated the associations between eight types of CRP and individual gut microbial communities. We provided a novel interpretative perspective for unraveling the intricate causal relationships between CRP and the gut microbiota.

## Methods

### Study design

**Figure 2:**
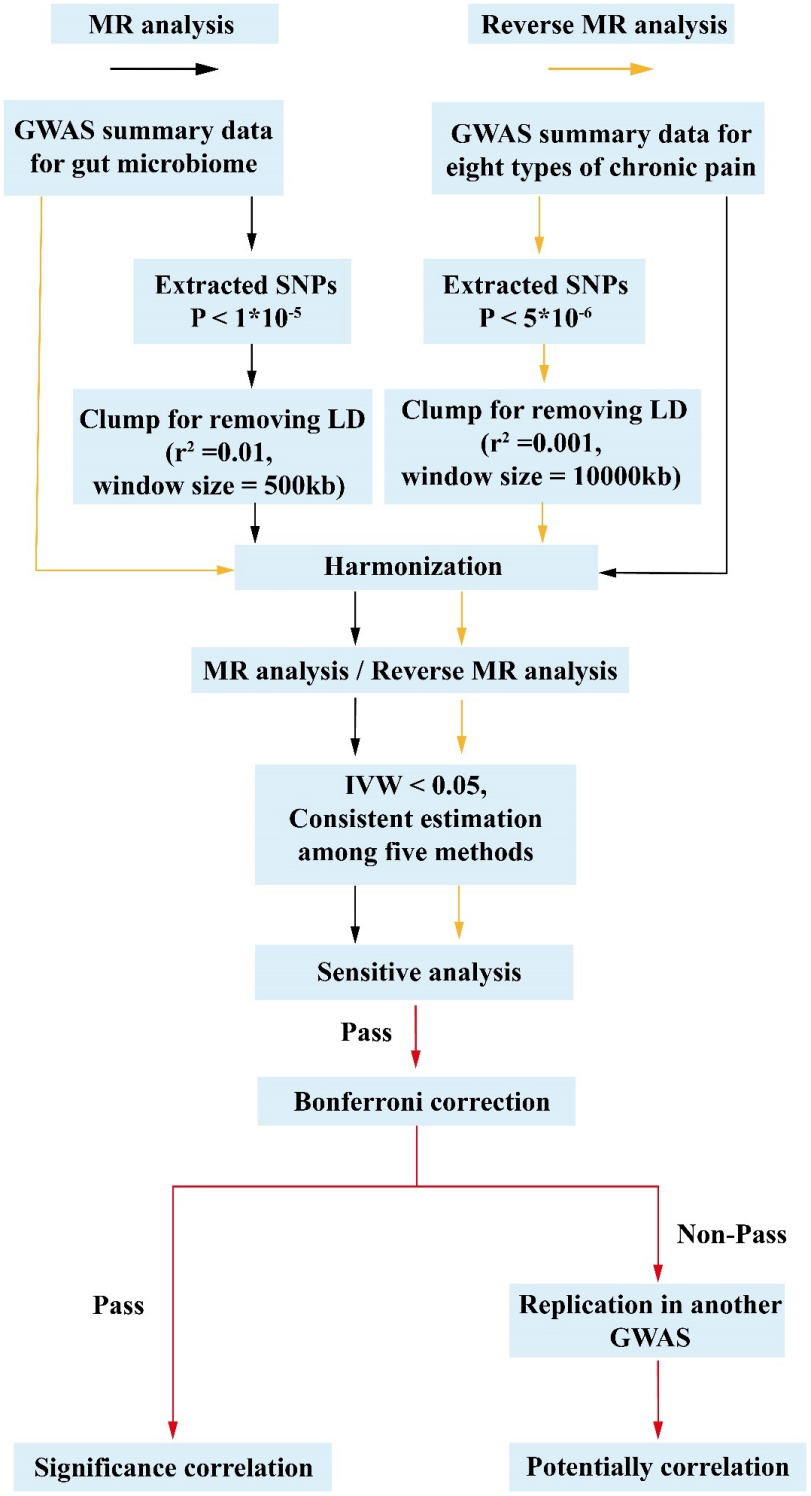
Flow chart of the study. In forward MR analysis, the instrumental variables of gut microbiotas were extracted in the following way: (1) *p* < 1 × 10^−5^; (2) *r*^2^ = 0.01, kb = 500. Five methods were used, and microbiotas with a *p*-value of IVW below 0.05, exhibiting consistent results of estimated value across the five analytical methods, were deemed to hold potential associations. Subsequently, an investigation into the presence of pleiotropy and heterogeneity among these potential floras was undertaken. Those floras displaying pleiotropic or heterogeneous characteristics were promptly eliminated. Then reverse MR analysis was performed, and different criteria were used for the screening of instrumental variables for AN (*p <* 5 × 10^−6^, *r*^2^ = 0.001, kb = 10,000). All potential flora were corrected by the Bonferroni test. The microbiotas passed the Bonferroni were considered significant. MR, Mendelian randomization; LD, linkage disequilibrium; GWAS, Genome Wide Association Study; SNPs, single-nucleotide polymorphisms; IVW, inverse-variance weighting.

In this study, SNPs strongly associated with exposure were used as IVs to explore the causal association between CRP and the gut microbiome. Due to allele frequency, disease prevalence may be different in different populations. Therefore, exposure and outcome need to be from the same race in MR analysis, which can rule out interference caused by population differences. At present, the publicly available intestinal flora databases are the main source of European populations, so this study selected European populations as the study population. The genome-wide association study (GWAS) summary data from two sets of gut microbiomes were employed (the MiBioGen Consortium [MC] and the Dutch Microbiome Project [DMP]). Initially, gut microbiomes were identified as the exposure, while eight distinct subtypes of CRP were designated as the outcomes for the MR analysis. Then, reverse MR analysis was employed to detect any reverse causal effect of CRP on gut microbiota. The flowchart is shown in Figure 2. The Strengthening the Reporting of Observational Studies in Epidemiology-Mendelian Randomization (STROBE-MR) checklist was used and interpreted in our study (Supplementary Table S1; Skrivankova et al., 2021).

### GWAS data of gut microbiome

First, GWAS summary data were obtained from MC (Kurilshikov et al., 2021). This study included 18,340 individuals from 24 cohorts (Kurilshikov et al., 2021). To the best of our knowledge, this GWAS summary data represent the largest dataset of gut microbiome available at the current stage. Three distinct regions (V1–V2, V3–V4, and V4) of the 16S rRNA gene were employed to analyze the gut microbiota composition (Kurilshikov et al., 2021). Genetic variants influencing the relative abundance of microbial taxa were identified using microbiota quantitative trait loci (mbQTL) mapping (Kurilshikov et al., 2021). Finally, 131 genera, 35 families, 20 orders, 16 classes, and 9 phyla were defined. Nonetheless, this GWAS data lack species-level gut microbiota data. Consequently, we integrated data from the DMP to augment our analysis (Lopera-Maya et al., 2022). The GWAS summary data for DMP comprised a comprehensive set of 5 phyla, 10 classes, 13 orders, 26 families, 48 genera, and 105 species. In this study, 7,738 individuals of European descent were included (Lopera-Maya et al., 2022). The detailed information is shown in Table 1.

**Table 1:** Details of GWAS summary data.

Trait	Sample size	Population	Data source
Gut microbiome			
MiBioGen Consortium	18,340	European (*N* = 13,266), American Hispanic/Latin (*N* = 1,097), East Asian (*N* = 811), Middle-Eastern (*N* = 481), African American (*N* = 114), Complex ancestry (*N* = 2,571)	https://www.mibiogen.org
Phylum			
Class			
Order			
Family			
Genus			
Dutch microbiome project	7,738	European (7,738 people)	https://dutchmicrobiomeproject.molgeniscloud.org/
Phylum			
Class			
Order			
Family			
Genus			
Species			
Chronic pain			https://gwas.mrcieu.ac.uk/datasets
Back pain	117,404	European	Ben Elsworth
	84,489	European	Neale lab
Facial pain	6,510	European	Neale lab
General pain	5,473	European	Neale lab
Headache	65,583	European	Neale lab
	91,269	European	Ben Elsworth
Hip pain	51,516	European	Ben Elsworth
Knee pain	97,889	European	Ben Elsworth
	70,992	European	Neale lab
Neck or shoulder pain	105,396	European	Ben Elsworth
	75,651	European	Neale lab
Stomach or abdominal pain	38,911	European	Ben Elsworth

The detailed information about the subtypes of chronic regional pain are found in Supplementary Table S2.

### GWAS data of chronic regional pain

The data for CRP were sourced from the IEU open GWAS project (v7.5.5-2023-08-09, *n* = 42,348). Pain persisting for more than 3 months was meticulously screened, encompassing general pain, headaches, facial pains, neck or shoulder pain, stomach or abdominal pain, back pain, knee pain, and hip pain. All individuals included in this study were of European ancestry, and comprehensive details regarding CRP are provided in Table 1 and Supplementary Table S2.

### The selection of IVs

For screening IVs, we rigorously adhere to the three fundamental assumptions of MR to ensure the accuracy of causality inference. SNPs, based on the criterion that a *p*-value of <1 × 10^−5^ was selected, were significantly associated with gut microbiota as potential eligible IVs (Yu et al., 2023). Then, to exclude the effect of linkage disequilibrium (LD), these SNPs, based on the European-based 1,000 Genome Projects reference panel, were clumped (*r*^2^ = 0.01, window size = 500 kb) (Xu et al., 2021). Finally, palindromic alleles were removed. For the reverse MR analysis, IVs of CRP were filtered. We chose a *p*-value of ≤5 × 10^−6^ as the criterion since the *p*-value of ≤5 × 10^−8^ was too strict. Then, the criterion of clump is reset (*r*^2^ = 0.001, window size = 10,000 kb) (Yu et al., 2023). Based on the following equation, we computed the *F*-values for each microbial community, which effectively mitigates potential bias from weak instruments (Xu et al., 2021):


$$F=R^{2}\times \frac{n-1-k}{\left(1-R^{2}\right)\times k}$$

*R*^2^ is to explain the exposure variance of the IVs, *n* is the sample size, and k is the number of IVs (Xu et al., 2021). When the F-value exceeds 10, it indicates the absence of a weak instrument bias (Pierce et al., 2011). Microbial communities with F-values less than 10 will be excluded.

### MR and reverse MR analysis

Five main MR analysis methods, including inverse-variance weighted (IVW), MR Egger, weighted median, simple mode, and weighted mode, were used to make causal inferences between gut microbiota and CRP. It was important to note that the accuracy of the IVW results depended on the validity of all IVs. Thus, the result of IVW could be affected by the pleiotropy and heterogeneity of IVs (Burgess et al., 2013). Nonetheless, IVW was still the most accurate method where these influences were absent (Chen et al., 2020). For evaluating horizontal pleiotropy and heterogeneity, we employed three methods, including the MR-Egger Intercept Test, Mendelian Randomization Pleiotropy RESidual Sum and Outlier (MR-PRESSO) global test, and Cochran’s Q-test (Greco et al., 2015; Long et al., 2023). For the MR-PRESSO method, it could detect the overall horizontal pleiotropy of IVs; furthermore, it could detect the abnormal SNPs that caused the existence of pleiotropy (Verbanck et al., 2018). The *p*-value of two methods (MR-Egger Intercept Test and MR-PRESSO) greater than 0.05 displayed that pleiotropy was absent. In addition, Cochran’s Q-test was employed to detect heterogeneity, which indicated non-heterogeneity with *p*-values above 0.05. Microbial communities exhibiting horizontal pleiotropy or heterogeneity will be excluded. Therefore, we excluded interference from heterogeneity and horizontal pleiotropy.

The positive result mainly depended on the method of IVW (Burgess et al., 2013). When the estimated value (beta) of the IVW aligns with that of the other four methods, even if only IVW < 0.05, we still consider this result to be meaningful (Wang et al., 2023). After harmonization, it is required to have a minimum match with at least three IVs for subsequent analysis. Therefore, if the matched SNPs are fewer than three, we refrain from conducting MR analysis, as this could lead to inaccurate results. Meanwhile, leave-one-out analysis was conducted to assess whether the estimate value of MR analysis was influenced or biased by a single SNP.

Additionally, to mitigate the impact of false positives in multiple tests, we employed Bonferroni correction, setting distinct statistically significant *p*-value thresholds based on microbial classification (phylum, class, order, family, and genus). For MC, the significant *p*-value thresholds were as follows: 0.00038 (genera), 0.0014 (families), 0.0025 (orders), 0.0031 (classes), and 0.0056 (phyla). For DMP, the significant *p*-values were as follows: 0.01 (phyla), 0.005 (classes), 0.0038 (orders), 0.0019 (families), 0.001 (genera), and 0.00048 (species). Subsequently, when the estimated directions of the five MR analysis methods are consistent and the IVW is below the threshold set by Bonferroni correction, the microbial community is considered to have yielded a positive result. To further disclose this potential correlation, microbial taxa that were associated with at least two different datasets in one phenotype, under the condition of IVW < 0.05, were considered to have a potentially more reliable and robust correlation. Furthermore, the analytical method of reverse MR analysis was the same as MR analysis. All analyses were accomplished by R.4.2.3^1^ and implemented by the packages TwoSampleMR (version 0.5.6), MR (version 0.7.0), and MRPRESSO (version 1.0).

## Results

### Instrumental variables

For MC, 231, 509, 1740, 289, and 126 SNPs associated with gut microbiota at different levels were identified (class, family, genus, order, and phylum; Supplementary Table S3). For DMP, 101, 257, 447, 118, 58, and 983 SNPs at different levels were also identified (class, family, genus, order, phylum, and species; Supplementary Table S4). For CRP, 111 SNPs were screened (Supplementary Table S5).

### Causal effects of MC on eight types of CRP

**Figure 3:**
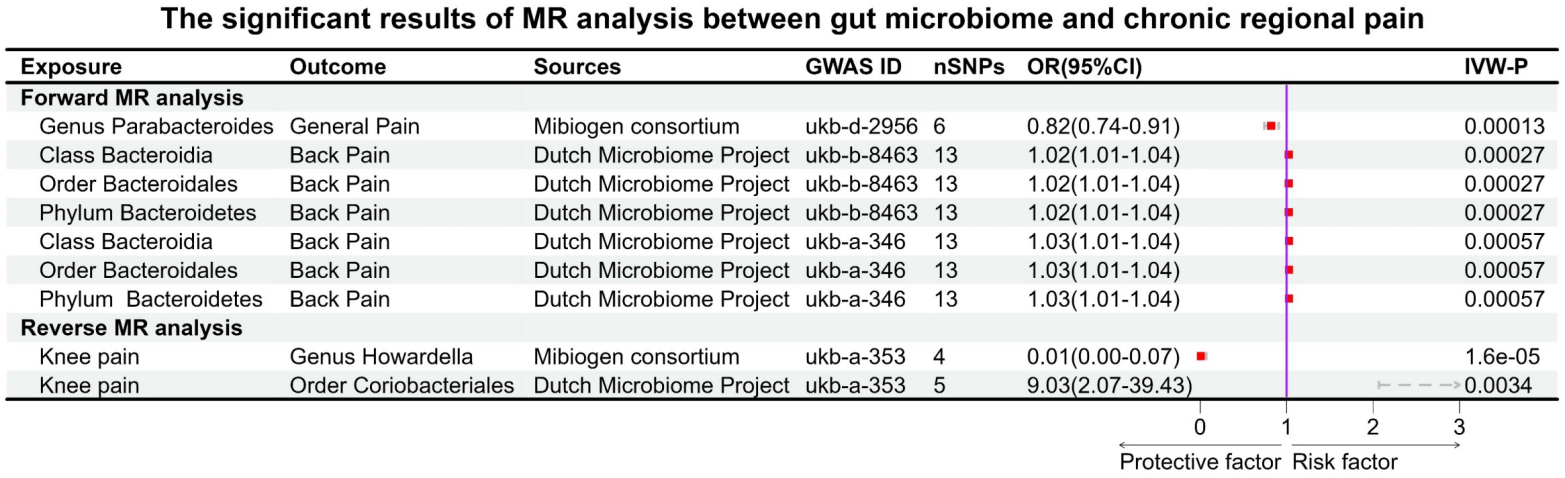
Significant results of MR analysis between gut microbiome taxa and chronic regional pain. Six gut microbial taxa had significant causal associations with three types of CRP (forward MR analysis: *Genus Parabacteroides* for general pain; *Class Bacteroidia*, *Order Bacteroidales*, and *Phylum Bacteroidetes* for back pain. Reverse MR analysis: knee pain for *Genus Howardella* and *Order Coriobacteriales*).

**Figure 4:**
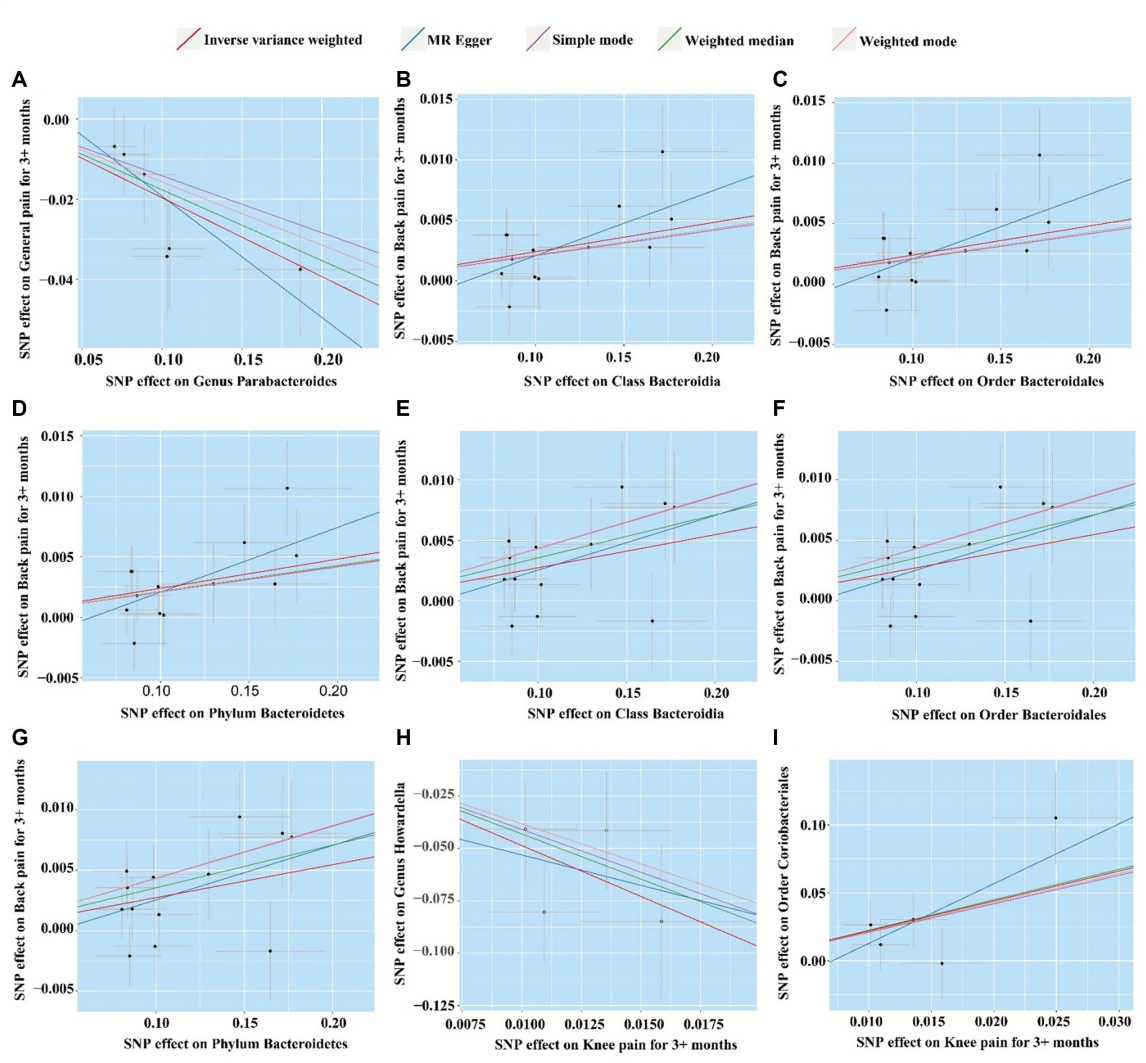
Scatter plot of five MR analysis methods for significant gut microbiome. **(A)** *Genus Parabacteroides* for general pain; **(B)** *Class Bacteroidia* for back pain (ukb-b-8463); **(C)** *Order Bacteroidales* for back pain (ukb-b-8463); **(D)** *Phylum Bacteroidetes* for back pain (ukb-b-8463); **(E)** *Class Bacteroidia* for back pain (ukb-a-346); **(F)** *Order Bacteroidales* for back pain (ukb-a-346); **(G)** *Phylum Bacteroidetes* for back pain (ukb-a-346); **(H)** Knee pain for *Order Coriobacteriales*; **(I)** Knee pain for *Genus Howardella.*

Before Bonferroni correction, our analysis revealed an association between 58 microbial taxa and CP (Supplementary Table S6). However, after applying the Bonferroni correction, only one was observed as a statistically significant microbial taxa (*Genus Parabacteroides* for general pain, OR: 0.82, 95% CI: 0.74–0.91, *p* = 0.00013, Figures 3; 4A).

### Causal effects of DMP on eight types of CRP

For the DMP, before Bonferroni correction, our analysis identified a correlation between 40 microbial taxa and CP (Supplementary Table S7). Three microbial taxa were still significantly associated with CP (back pain) after Bonferroni correction, including *Class Bacteroidia* (OR: 1.02, 95% CI:1.01–1.04, *p* = 0.00027), *Order Bacteroidales* (OR:1.02, 95% CI:1.01–1.04, *p* = 0.00027), and *Phylum Bacteroidetes* (OR:1.02, 95% CI:1.01–1.04, *p* = 0.00027). This result was replicated across the two GWAS databases for back pain (Figures 3, 4B–G).

### Causal effects of eight types of CRP on gut microbiota (MC and DMP)

In the GWAS data for MC, we identified 58 gut microbiotas potentially associated with CP, while in the DMP GWAS data, there were 48 gut microbiotas with potential links to CP (Supplementary Tables S8, S9). During the reverse MR analysis, only two SNPs, after harmonization, exhibited a significant association between facial pain and the gut microbiota (MC). Thus, according to our criteria, the analysis between gut microbiota (MC) and facial pain was unable to be executed. After Bonferroni correction, two microbiotas had a reverse causal effect on CRP (knee pain), as shown in Figure 3. *Genus Howardella* (OR:0.01, 95% CI:0.00–0.07, *p* = 1.6 × 10^−5^) was a protective factor for knee pain. For DMP, after Bonferroni correction, *Order Coriobacteriales* (OR:9.03, 95% CI:2.07–39.43, *p* = 0.0034) was found as a risk factor for knee pain (Figures 3, 4H,I).

### Potential correlation between gut microbiota and CRP

**Figure 5:**
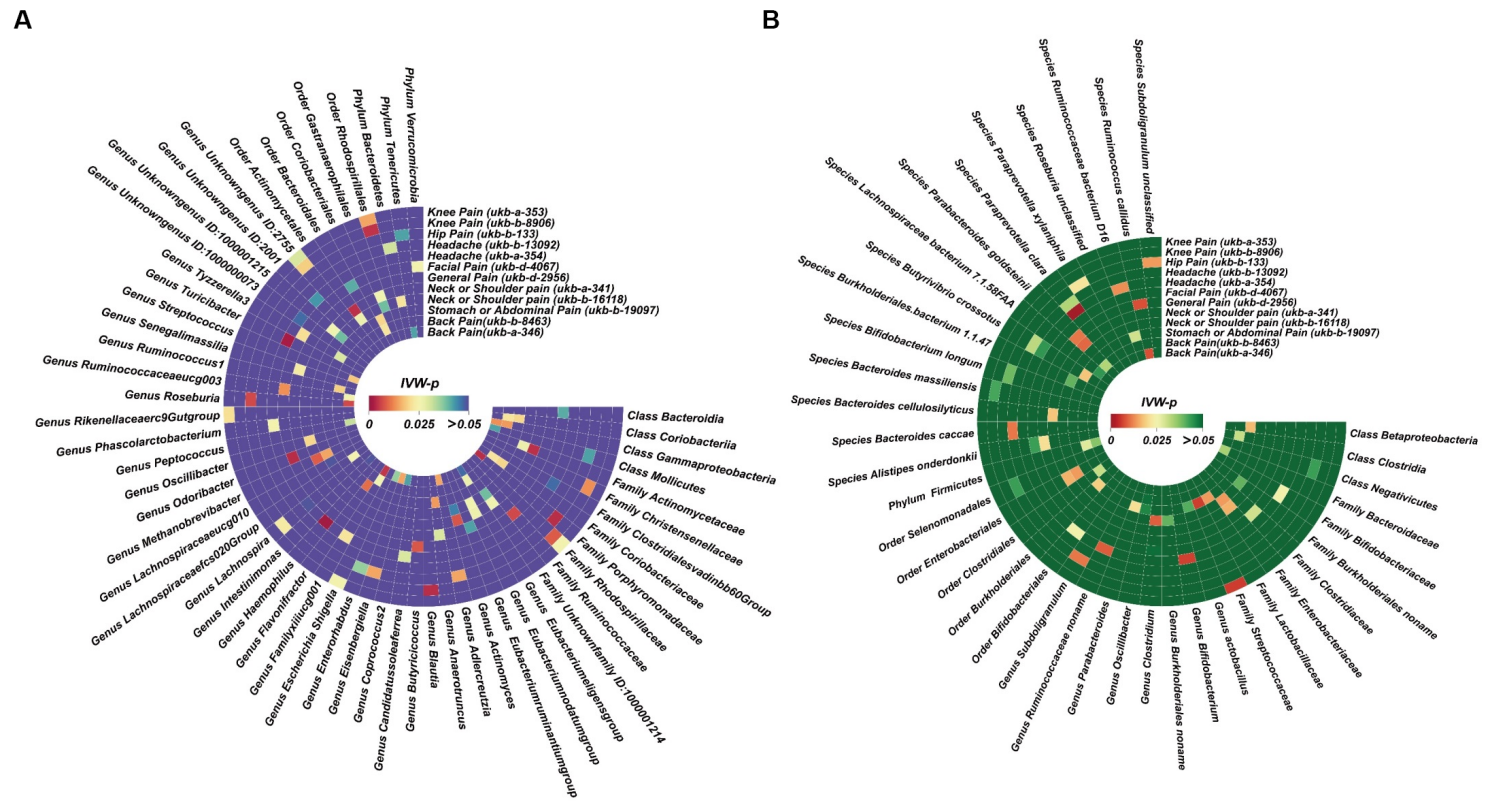
Potential causal effect of the gut microbiome on chronic regional pain. **(A)** Results of forward MR analysis (IVW) between the MiBioGen Consortium and eight chronic regional pains. **(B)** Results of forward MR analysis (IVW) between the Dutch Microbiome Project and eight chronic regional pains.

**Figure 6:**
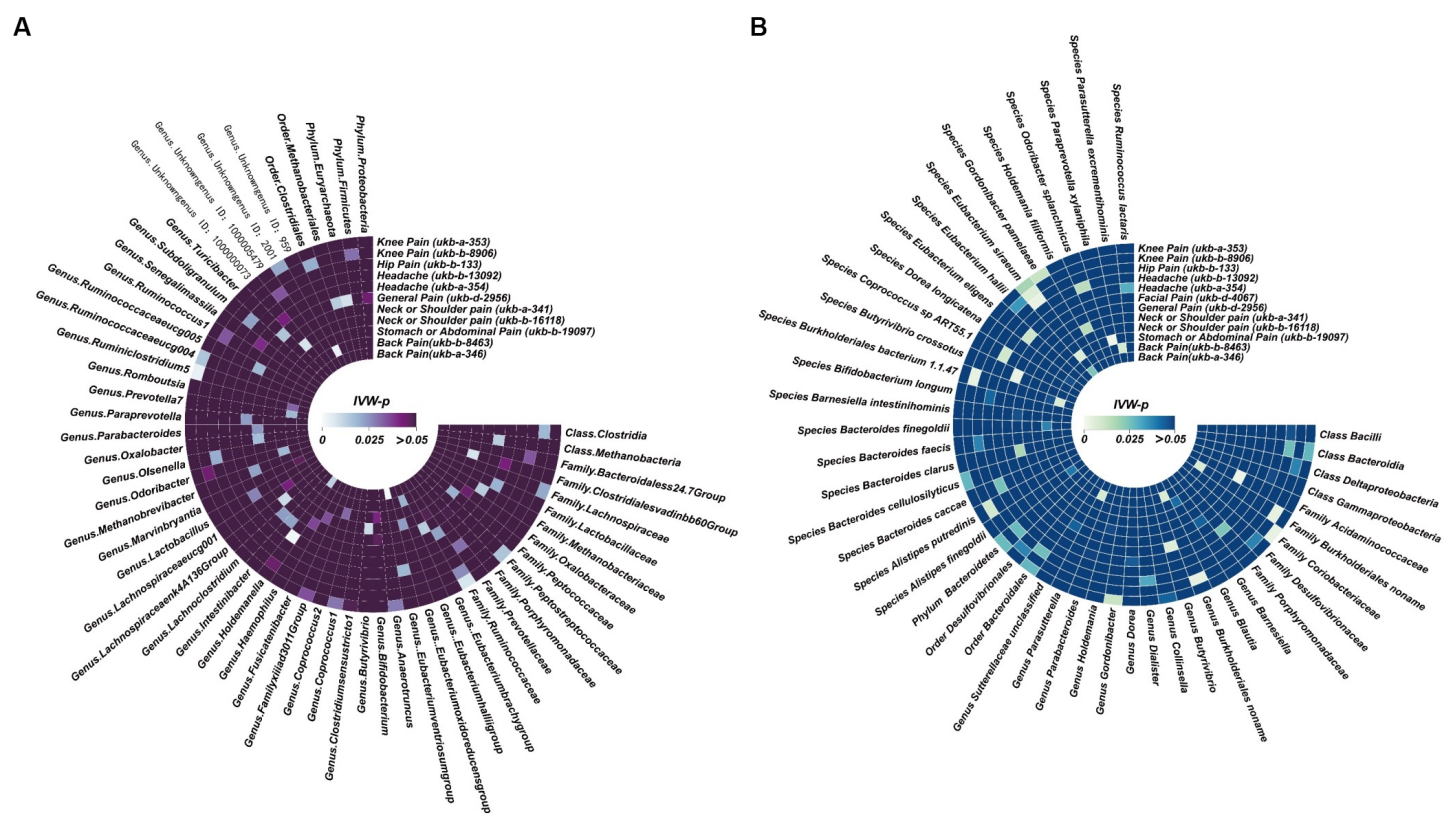
Potential causal effect of chronic regional pains on the gut microbiome. **(A)** Results of reverse MR analysis (IVW) between the MiBioGen Consortium and seven chronic regional pains. Facial pain did not meet the requirements we set in the MR analysis. Thus, we did not analyze the causal effect of facial pain on the MiBioGen Consortium. **(B)** Results of reverse MR analysis (IVW) between the Dutch Microbiome Project and eight chronic regional pains.

**Figure 7:**
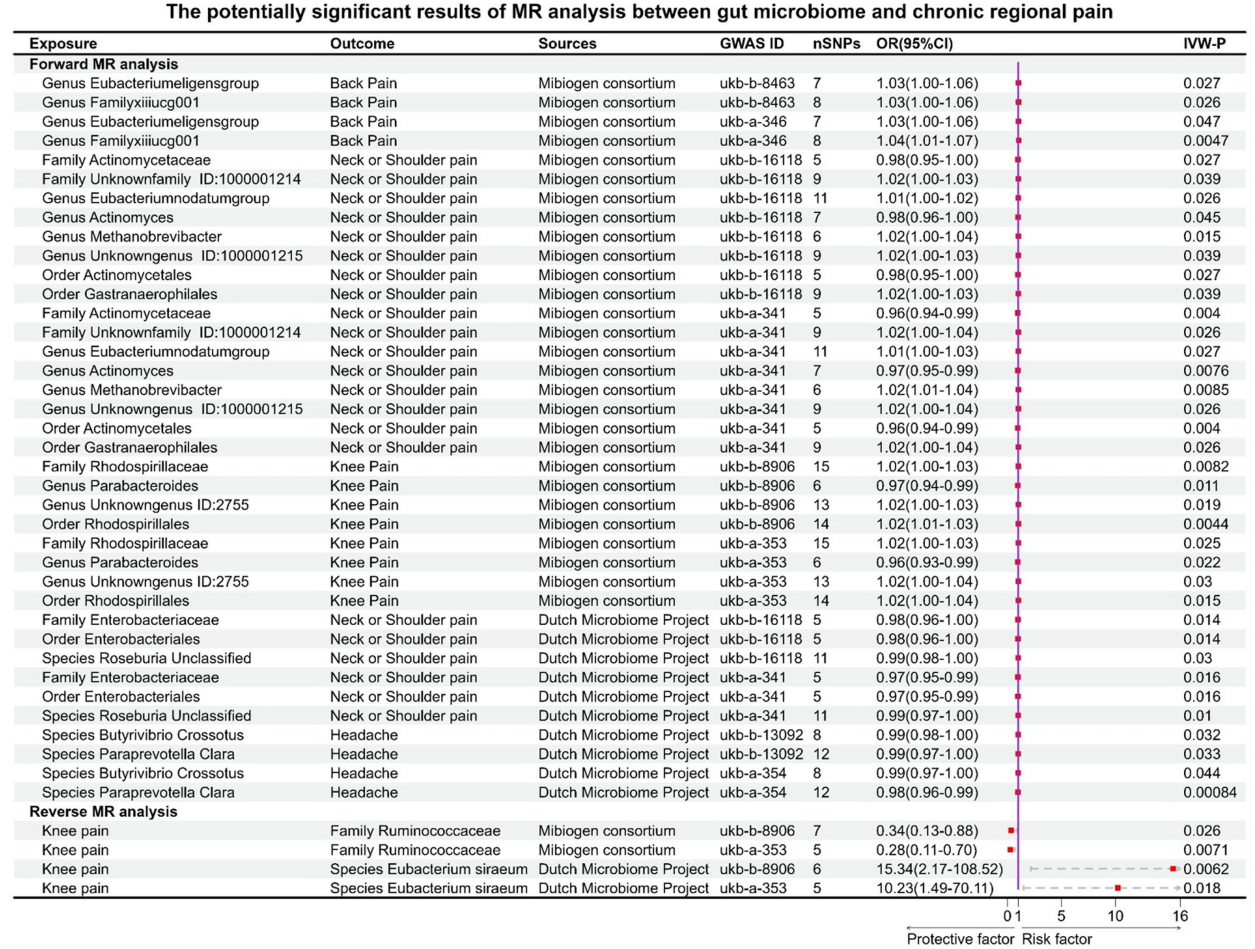
Potential causal effect between the gut microbiome and chronic regional pains. This forest plot is used to illustrate the 19 microbial taxa potentially associated with chronic regional pain.

We also identified, under the condition of IVW < 0.05, several potentially significant microbial taxa that are linked to CP (Figures 5A,B, 6A,B). Further analysis revealed that 19 microbial taxa showed a correlation with both distinct datasets (Figure 7).

For back pain, *Genus Eubacterium eligens group* (GWASID: ukb-b-8463, OR:1.03, 95% CI:1.00–1.06, *p* = 0.027; GWSID: ukb-a-346, OR:1.03, 95CI%: 1.00–1.06, *p* = 0.026) and *Genus FamilyXIIIUCG001* (GWASID: ukb-b-8463, OR:1.03, 95% CI: 1.00–1.06, *p =* 0.026; GWASID: ukb-a-346, OR:1.04, 95% CI: 1.01–1.07, *p =* 0.0047) were potential risk factors (Figure 7).

For neck or shoulder pain, the different gut microbiomes also display that association (Figure 7). In gut microbiome from MC, the results showed that *Family Unknown family ID:1000001214* (GWASID: ukb-b-16118, OR:1.02, 95% CI: 1.00–1.03, *p =* 0.039; GWASID: ukb-a-341, OR:1.02, 95% CI: 1.00–1.04, *p =* 0.026), *Genus Eubacterium nodatum group* (GWASID: ukb-b-16118, OR:1.01, 95% CI: 1.00–1.02, *p =* 0.026; GWASID: ukb-a-341, OR:1.01, 95% CI: 1.00–1.03, *p =* 0.027), *Genus Methanobrevibacter* (GWASID: ukb-b-16118, OR:1.02, 95% CI: 1.00–1.04, *p =* 0.015; GWASID: ukb-a-341, OR:1.02, 95% CI: 1.01–1.04, *p =* 0.0085), and *Genus Unknown genus ID:1000001215* (GWASID: ukb-b-16118, OR:1.02, 95% CI: 1.00–1.03, *p =* 0.039; GWASID: ukb-a-341, OR:1.02, 95% CI: 1.00–1.04, *p =* 0.026) were risk factors. *Family Actinomycetaceae* (GWASID: ukb-b-16118, OR:0.98, 95% CI: 0.95–1.00, *p =* 0.027; GWASID: ukb-a-341, OR:0.96, 95% CI: 0.94–0.99, *p =* 0.004), *Genus Actinomyces* (GWASID: ukb-b-16118, OR:0.98, 95% CI: 0.96–1.00, *p =* 0.045; GWASID: ukb-a-341, OR:0.97, 95% CI: 0.95–0.99, *p =* 0.0076), *Order Gastranaerophilales* (GWASID: ukb-b-16118, OR: 1.02, 95% CI: 1.00–1.03, *p* = 0.039; GWASID: ukb-a-341, OR: 1.02, 95% CI: 1.00–1.04, *p* = 0.026), and *Order Actinomycetales* (GWASID: ukb-b-16118, OR:0.98, 95% CI: 0.95–1.00, *p =* 0.027; GWASID: ukb-a-341, OR:0.96, 95% CI: 0.94–0.99, *p =* 0.004) were potentially protective factors for neck or shoulder pain. In gut microbiome from DMP, we found that genetically predicted neck or shoulder pain was potentially related to a low abundance of *Species Roseburia Unclassified* (GWASID: ukb-b-16118, OR:0.99, 95% CI: 0.98–1.00, *p =* 0.03; GWASID: ukb-a-341, OR:0.99, 95% CI: 0.97–1.00, *p =* 0.01), *Family Enterobacteriaceae* (GWASID: ukb-b-16118, OR:0.98, 95% CI:0.96–1.00, *p =* 0.014; GWASID: ukb-a-341, OR:0.97, 95% CI:0.95–0.99, *p =* 0.016), and *Order Enterobacteriales* (GWASID: ukb-b-16118, OR:0.98, 95% CI: 0.96–1.00, *p =* 0.014; GWASID: ukb-a-341, OR:0.97, 95% CI: 0.95–0.99, *p =* 0.016).

For knee pain, we found that genetically predicted knee pain was potentially related to higher abundance of *Family Rhodospirillaceae* (GWASID: ukb-b-8906, OR:1.02, 95% CI: 1.00–1.03, *p =* 0.0082; GWASID: ukb-a-353, OR:1.02, 95% CI: 1.00–1.03, *p =* 0.025), *Genus Unknown genus ID:2755* (GWASID: ukb-b-8906, OR:1.02, 95% CI: 1.00–1.03, *p =* 0.019; GWASID: ukb-a-353, OR:1.02, 95% CI: 1.00–1.04, *p =* 0.03), and *Order Rhodospirillales* (GWASID: ukb-b-8906, OR:1.02, 95% CI: 1.01–1.03, *p =* 0.0044; GWASID: ukb-a-353, OR:1.02, 95% CI: 1.00–1.04, *p =* 0.015). Notably, we found that genetically predicted knee pain was potentially related to a low abundance of *Genus Parabacteroides* (GWASID: ukb-b-8906, OR:0.97, 95% CI:0.94–0.99, *p =* 0.011; GWASID: ukb-a-353, OR:0.96, 95% CI:0.93–0.99, *p =* 0.022).

For headache, *Species Butyrivibrio crossotus* (GWASID: ukb-b-13092, OR:0.99, 95% CI: 0.98–1.00, *p =* 0.032; GWASID: ukb-a-354, OR:0.99, 95% CI: 0.97–1.00, *p =* 0.044), *Species Paraprevotella Clara* (GWASID: ukb-b-13092, OR:0.99, 95% CI: 0.97–1.00, *p =* 0.033; GWASID: ukb-a-354, OR:0.98, 95% CI: 0.96–0.99, *p =* 0.00084).

In reverse MR analysis, several microbial taxa were potentially influenced by CRP (Figures 6A,B). Further analysis of this association revealed that the *Family Ruminococcaceae* (GWASID: ukb-b-8906, OR:0.34, 95% CI: 0.13–0.88, *p =* 0.026; GWASID: ukb-a-353, OR:0.28, 95% CI: 0.11–0.70, *p =* 0.0071) was potentially correlated with both of knee’s distinct datasets (Figure 7). *Species Eubacterium siraeum* (GWASID: ukb-b-8906, OR:15.34, 95% CI: 2.17–108.52, *p =* 0.0062; GWASID: ukb-a-353, OR:10.23, 95% CI: 1.49–70.11, *p =* 0.018) also exhibited a correlation with both of knee’s distinct datasets (Figure 7).

### Sensitivity analysis

**Figure 8:**
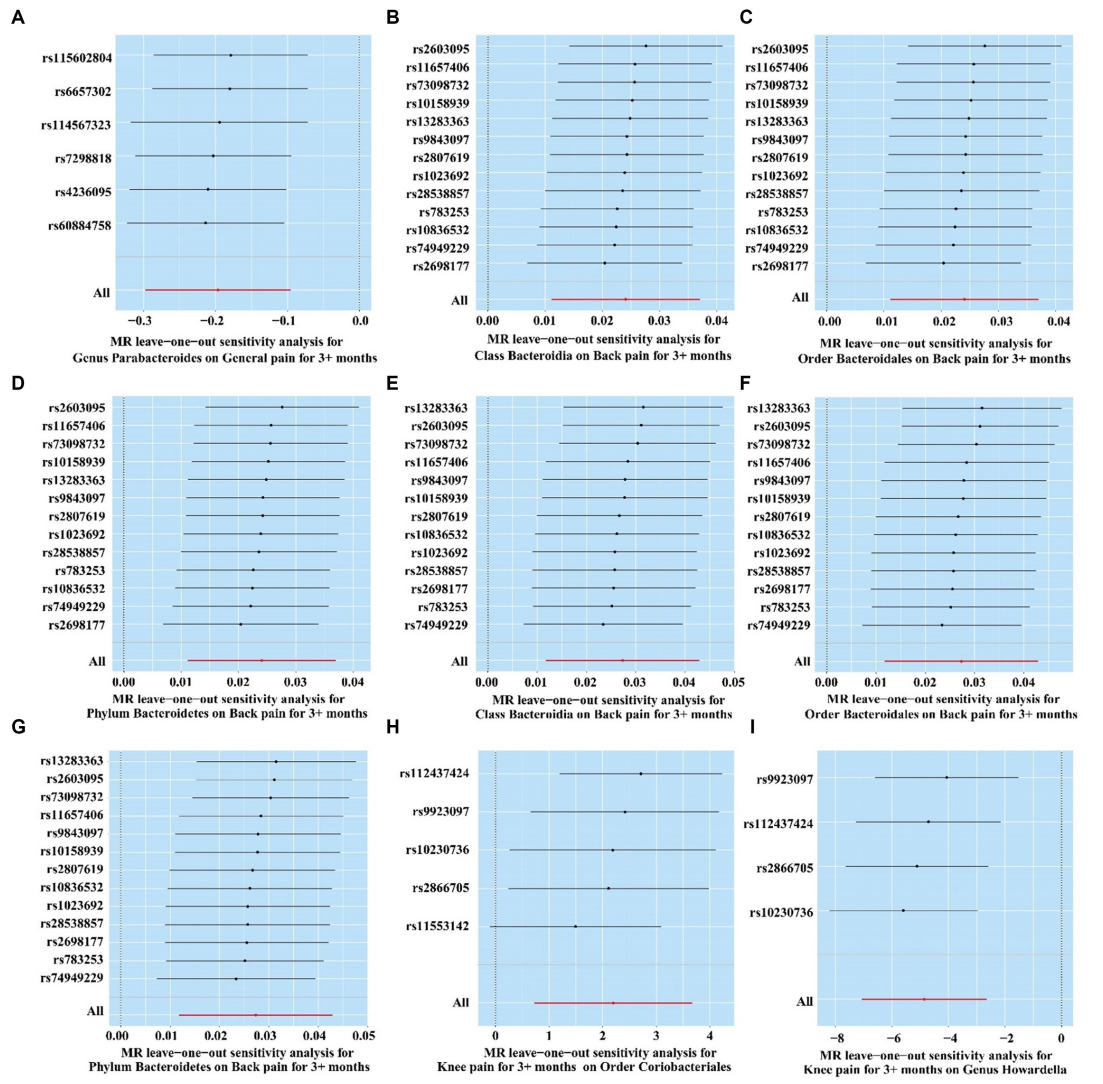
Leave-one-out analysis of significant gut microbiome. **(A)** *Genus Parabacteroides* for general pain; **(B)** *Class Bacteroidia* for back pain (ukb-b-8463); **(C)** *Order Bacteroidales* for back pain (ukb-b-8463); **(D)** *Phylum Bacteroidetes* for back pain (ukb-b-8463); **(E)** *Class Bacteroidia* for back pain (ukb-a-346); **(F)** *Order Bacteroidales* for back pain (ukb-a-346); **(G)** *Phylum Bacteroidetes* for back pain (ukb-a-346); **(H)** Knee pain for *Order Coriobacteriales*; **(I)** Knee pain for *Genus Howardella.*

The result of MR-PRESSO and MR Egger regression did not display horizontal pleiotropy among the significant and potential microbiota (Table 2; Supplementary Tables S10–S13). In addition, the result of Cochran’s Q-test also did not indicate heterogeneity (Table 2; Supplementary Tables S10–S13). Moreover, there is no single SNP, in leave-one-out analysis, that strongly violates the overall effect between the gut microbiome and CRP (Figure 8). Furthermore, all F-statistical values of significant and potential gut microbiotas exceeded 10 (Table 2; Supplementary Tables S10–S13).

**Table 2:** Significant results of sensitivity analysis.

Exposure	Outcome	Source	GWAS ID	MR Egger intercept test	MR-PRESSO Global test	Cochran’s Q-test	*R* ^2^	*F*-value
Forward MR analysis								
Genus Parabacteroides	General pain	MC	ukb-d-2956	0.513	0.855	0.787	0.019	32.88
Class Bacteroidia	Back pain	DMP	ukb-a-346	0.549	0.445	0.415	0.037	23.08
Order Bacteroidales	Back pain	DMP	ukb-a-346	0.549	0.446	0.415	0.037	23.08
Phylum bacteroidetes	Back pain	DMP	ukb-a-346	0.551	0.445	0.415	0.037	23.07
Class Bacteroidia	Back pain	DMP	ukb-b-8463	0.236	0.584	0.540	0.037	23.08
Order Bacteroidales	Back pain	DMP	ukb-b-8463	0.236	0.584	0.540	0.037	23.08
Phylum bacteroidetes	Back pain	DMP	ukb-b-8463	0.236	0.584	0.539	0.037	23.07
Reverse MR analysis								
Knee pain	Genus Howardella	MC	ukb-a-353	0.798	0.649	0.552	0.002	22.77
Knee pain	Order Coriobacteriales	DMP	ukb-a-353	0.450	0.405	0.331	0.002	22.98

MR, Mendelian randomization; MC, MiBioGen Consortium; DMP, Dutch Microbiome Project.

## Discussion

To our knowledge, this is the first systematic study that utilizes MR analysis to detect the causal effect between gut microbiota and eight types of CRP. While previous studies have explored the association between CP and gut microbiotas, these studies could be influenced by potential confounding factors, such as socioeconomic status and dietary habits (Davies et al., 2018). Additionally, these studies did not delve into the relationship between individual microbial taxa and CRP. Our study bridges this gap and provides new insights from a new perspective.

Our research reveals that *Genus Parabacteroides* is considered a protective factor against chronic widespread pain. It is worth noting that recent studies have suggested that *Genus Parabacteroides* may represent a novel type of gut probiotic (Wu, 2023). It has therapeutic effects on tumors, metabolic diseases, and inflammatory conditions (Wu, 2023). A recent study also displayed a reduction in the abundance of *Genus Parabacteroides* in the gut of rheumatoid arthritis patients. *Genus Parabacteroides* may alleviate rheumatoid arthritis by directly or indirectly affecting the differentiation of T helper 17 cells through secondary bile acids. Interestingly, rheumatoid arthritis patients often experience CP (Sun et al., 2023). *Class Bacteroidia*, *Order Bacteroidales*, and *Phylum Bacteroidetes* have not been reported to be associated with CRP. Our study, for the first time, demonstrated their relationship with chronic back pain. Importantly, these three microbial taxa passed the Bonferroni correction and showed consistency across two different GWAS datasets for the phenotype of persistent back pain. Therefore, we consider this conclusion to be highly reliable. Perhaps regulating the abundance of these three microbial taxa in the gut could be a means of preventing back pain. In reverse MR analysis, we found that knee pain leads to a higher abundance of *Genus Howardella*, while the abundance of *Order Coriobacteriales* decreases. Interestingly, previous research did not uncover the associations between these two microbial taxa and chronic knee pain.

In addition to significant microbiota, we also identified 19 microbial taxa associated with CRP. We observed some intriguing phenomena. Previous research has suggested that the *Genus Eubacterium nodatum group* and *Genus Methanobrevibacter* may be associated with abdominal pain (Kumpitsch et al., 2021; Jiang et al., 2022). In fecal samples from patients with nodular lymphoid hyperplasia, the abundance of the *Genus Eubacterium nodatum group*, compared to the control group, was significantly higher, and nodular lymphoid hyperplasia is often accompanied by persistent abdominal pain (Jiang et al., 2022). The *genus Methanobrevibacter* is considered a major contributor to methane production in the human gut. Individuals with high methane levels in their guts can have *Genus Methanobrevibacter* abundances up to 1,000 times higher than normal individuals (Kumpitsch et al., 2021). Elevated methane in the gut has been linked to symptoms such as abdominal pain, bloating, and constipation (Kumpitsch et al., 2021). However, our study revealed that an increased abundance of these microbial groups is more likely to be associated with neck and shoulder pain. Additionally, our study unearthed an intriguing phenomenon. We observed that chronic knee pain has an impact on four distinct gut microbiotas, including *Genus Howardella*, *Order Coriobacteriales, Family Ruminococcaceae, and Species Eubacterium siraeum*. Surprisingly, we did not uncover any supporting evidence for this in the existing literature. This could potentially be attributed to various factors, including mediating effects such as dietary habits, sleep patterns, and other contributing factors. Nevertheless, another paradoxical observation is the absence of alterations in the gut microbiota associated with other forms of CRP. This suggests the possibility of additional mechanisms at play in influencing the gut microbiota beyond those related to chronic knee pain. One plausible hypothesis is that these microbial communities participate in the occurrence and development of CP through boron metabolism. Recent studies have indicated that prebiotic boron complexes (PBCs), rich in boron, can engage in signal transmission between the host and microbial communities, maintaining a balance between beneficial and harmful bacteria in the gut (Fang et al., 2021). Simultaneously, they contribute to improving the permeability of the intestinal mucosa, preventing inflammatory substances from entering the bloodstream, and thereby impeding the onset of low-intensity chronic inflammation (Bita et al., 2023). Further research could shed light on their underlying mechanisms.

We must acknowledge several limitations in our study. First, to secure a sufficient number of IVs, we employed different selection criteria for gut microbiota and CRP. Additionally, we relaxed the screening threshold for CRP (*p* < 5 × 10^−6^), compared to the more stringent standard commonly used in the literature (*p* < 5 × 10^−8^). This decision may have potentially weakened the first assumption of MR. Second, the GWAS summary data in our study are derived from European ancestry, including both gut microbiota and CP data. Therefore, our findings for other ethnic groups might not be suitable. Third, while most of the GWAS data for MC are from individuals of European ancestry, the racial composition of the two datasets we employed is not entirely congruent. This incongruence may introduce some level of inconsistency in LD correlations. Fourth, in light of the limited availability of GWAS data for certain site-specific CP conditions, our established criteria were unable to reveal a potential association between microbial communities and the corresponding type of pain. Due to the limitations of MR studies, it is also not clear whether the gut microbiome and CRP are involved in the regulation of disease through certain substances acting as vectors. In addition, the description of pain data does make it impossible to identify the specific cause of pain, which may have a potential impact on our results. Finally, we were unable to access detailed individual-level information such as age, gender, inclusion, and exclusion criteria. Consequently, we could not conduct further stratified analyses.

Despite these limitations, we believe that our study provides novel insights into the potential associations between the gut microbiota and site-specific CP. It lays a solid foundation for future research endeavors, even as we acknowledge the need for caution in generalizing our conclusions, particularly to diverse populations and under varying circumstances.

In conclusion, through the utilization of publicly accessible genetic databases, we have uncovered reciprocal causal connections between particular gut microbiotas and CRP. Our discoveries strongly imply that there could be a direct correlation between distinct CRP and specific gut microbiota compositions. By modulating the biological abundance of the corresponding microbiota, precise treatment goals may be achieved.

## Data availability statement

The original contributions presented in the study are included in the article/Supplementary material, further inquiries can be directed to the corresponding authors.

## Author contributions

Q-AX: Data curation, Formal analysis, Investigation, Writing – original draft. LQ: Methodology, Software, Writing – review & editing. JY: Resources, Visualization, Writing – review & editing. Y-TH: Formal analysis, Resources, Writing – review & editing. L-FA: Formal analysis, Methodology, Software, Writing – review & editing. D-CW: Conceptualization, Funding acquisition, Writing – review & editing. XX: Funding acquisition, Project administration, Supervision, Writing – original draft. X-LZ: Conceptualization, Supervision, Validation, Writing – review & editing.

## Footnotes

^1^http://www.R-project.org
