# Supplementary material for: The causality between gut microbiome and chronic regional pain: a Mendelian randomization analysis
Source: Front Microbiol. 2024 Feb 29;15:1329521. doi: 10.3389/fmicb.2024.1329521 (PMC10938595; doi:10.3389/fmicb.2024.1329521)
Supplement: Supplementary file 1 [file Table_1.DOCX]

**Table S1: STROBE-MR checklist of recommended items to address in reports of Mendelian randomization studies**

| **Item No.** | **Section** | **Checklist item** | **Page No.** | **Relevant text from manuscript** |
| --- | --- | --- | --- | --- |
| 1 | **TITLE and ABSTRACT** | Indicate Mendelian randomization (MR) as the study’s design in the title and/or the abstract if that is a main purpose of the study | 1 | The causality between gut microbiome and chronic regional pain: a two samples bi-direction Mendelian randomization analysis |
|  | **INTRODUCTION** |  |  |  |
| 2 | **Background** | Explain the scientific background and rationale for the reported study. What is the exposure? Is a potential causal relationship between exposure and outcome plausible? Justify why MR is a helpful method to address the study question | 5 | Emerging evidence hints at the potential and pivotal role of gut microbiota in various types of CP, including abdominal pain, headaches, inflammatory pain, chronic widespread musculoskeletal pain, and neuropathic discomfort. Nevertheless, currently, there is a lack of comprehensive causal association analysis between gut microbiota and chronic regional pain (CRP), such as neck pain, shoulder pain. In addition, ongoing research is susceptible to diverse confounding elements, spanning factors such as nutritional status, sleep cycles, and physical activity. Furthermore, the intricate associations between distinct gut microbiota compositions and CP further complexify the exploration of their interplay. |
| 3 | **Objectives** | State specific objectives clearly, including pre-specified causal hypotheses (if any). State that MR is a method that, under specific assumptions, intends to estimate causal effects | 5-6 | ased on the above background, we propose a hypothesis: there is an association between gut microbiota and CRP. Consequently, we employed Mendelian randomization (MR) to comprehensive investigate this association. |
|  | **METHODS** |  |  |  |
| 4 | **Study design and data sources** | Present key elements of the study design early in the article. Consider including a table listing sources of data for all phases of the study. For each data source contributing to the analysis, describe the following: |  |  |
|  | a) | Setting: Describe the study design and the underlying population, if possible. Describe the setting, locations, and relevant dates, including periods of recruitment, exposure, follow-up, and data collection, when available. | 6-7  Table 1 and Supplementary table 2 | Detailed information can be found in the Table 1 and Supplementary table 1 |
|  | b) | Participants: Give the eligibility criteria, and the sources and methods of selection of participants. Report the sample size, and whether any power or sample size calculations were carried out prior to the main analysis | 6-7  Table 1 and Supplementary table 2 | Detailed information can be found in the Table 1 and Supplementary table 1 |
|  | c) | Describe measurement, quality control and selection of genetic variants | 7-8  Table 1 and Supplementary table 2 | Detailed information can be found in the Table 1 and Supplementary table 1.  The criterion for selecting IVs is as follows……. |
|  | d) | For each exposure, outcome, and other relevant variables, describe methods of assessment and diagnostic criteria for diseases | 7  Table 1 and Supplementary table 2 | Detailed information can be found in the Table 1 and Supplementary table 1.  The GWAS summary data of pain persisting for more than 3 months were meticulously screened…… |
|  | e) | Provide details of ethics committee approval and participant informed consent, if relevant | No applicable | The data analyzed in this secondary study is publicly available from existing, published GWASs and therefore the ethical approval and informed consent have been obtained by all original studies. |
| 5 | **Assumptions** | Explicitly state the three core IV assumptions for the main analysis (relevance, independence and exclusion restriction) as well assumptions for any additional or sensitivity analysis | 5, 7 | MR analysis is based on three basic assumptions: (1) IVs must be strongly correlated with exposure; (2) IVs cannot be correlated with confounding factors; (3) IVs can only affect outcomes through exposure factors  When selecting IVs, we rigorously adhere to the three fundamental assumptions of MR to ensure the accuracy of causality inference. |
| 6 | **Statistical methods: main analysis** | Describe statistical methods and statistics used |  |  |
|  | a) | Describe how quantitative variables were handled in the analyses (i.e., scale, units, model) | 6-7  Table 1 and Supplementary table 2 | Supplementary table 1 provides a detailed summary of chronic regional pain within the IEU Open GWAS Project |
|  | b) | Describe how genetic variants were handled in the analyses and, if applicable, how their weights were selected | 7 | SNPs, significantly associated with each gut microbiota, were selected (the P value of SNPs < 1*10-5) as the potential eligible IVs; (2) These SNPs were clumped for excluding the effect of linkage disequilibrium (r2=0.01, window size= 500kb) based on the European-based 1,000 Genome Projects reference panel; (3) palindromic alleles were removed. Then, for the reverse MR analysis, IVs of CRP were filtered. However, P ≤ 5*10-8 is so strict that small SNPs were filtered. Thus, we choose P ≤ 5*10-6 as the criterion and clump is reset (r2=0.001, window size= 10000kb) |
|  | c) | Describe the MR estimator (e.g. two-stage least squares, Wald ratio) and related statistics. Detail the included covariates and, in case of two-sample MR, whether the same covariate set was used for adjustment in the two samples | 6-7 | The list of covariates varies between original GWASs, but always included sex. Details can be found in the Supplementary table 1 |
|  | d) | Explain how missing data were addressed | No applicate | We found no relevant information contained in IEU open GWAS |
|  | e) | If applicable, indicate how multiple testing was addressed | 9 | We applied the Bonferroni correction method to adjust the P-values, mitigating the potential for false positives (P < 0.05/N, N represents the effective number of independent bacterial taxa at the specific taxonomic level). |
| 7 | **Assessment of assumptions** | Describe any methods or prior knowledge used to assess the assumptions or justify their validity | 8 | Different approaches yield valid evidence under different assumptions: (1) For IVW method, it estimates the causal effect of exposure on the outcome by aggregating the ratio estimates for each SNP…… |
| 8 | **Sensitivity analyses and additional analyses** | Describe any sensitivity analyses or additional analyses performed (e.g. comparison of effect estimates from different approaches, independent replication, bias analytic techniques, validation of instruments, simulations) | 8 | Different approaches yield valid evidence under different assumptions: (1) For IVW method, it estimates the causal effect of exposure on the outcome by aggregating the ratio estimates for each SNP…… |
| 9 | **Software and pre-registration** |  |  |  |
|  | a) | Name statistical software and package(s), including version and settings used | 10 | All data processing and analysis were accomplished by R software (R.4.2.3; http://www.R-project.org). The R packages used in study are TwoSampleMR (version: 0.5.6), MendelianRandomization (version: 0.7.0), and MRPRESSO (version: 1.0). |
|  | b) | State whether the study protocol and details were pre-registered (as well as when and where) | No Applicable | This is a secondary analysis based on summary statistics from existing, published studies. |
|  | **RESULTS** |  |  |  |
| 10 | **Descriptive data** |  |  |  |
|  | a) | Report the numbers of individuals at each stage of included studies and reasons for exclusion. Consider use of a flow diagram | 6-7 | Detailed information can be found in the Table 1 and Supplementary table 1. |
|  | b) | Report summary statistics for phenotypic exposure(s), outcome(s), and other relevant variables (e.g. means, SDs, proportions) | 7 | Detail described shown in the part of GWAS data of Gut microbiome and GWAS data of Chronic regional pain |
|  | c) | If the data sources include meta-analyses of previous studies, provide the assessments of heterogeneity across these studies | No Applicable | We didn’t use the meta-analysis of studies |
|  | d) | For two-sample MR:  i.  Provide justification of the similarity of the genetic variant-exposure associations between the exposure and outcome samples  ii.  Provide information on the number of individuals who overlap between the exposure and outcome studies | No page | These GWAS samples are sourced from various research institutions, thus largely avoiding sample overlap. The primary population source for these GWAS is of European descent. |
| 11 | **Main results** |  |  |  |
|  | a) | Report the associations between genetic variant and exposure, and between genetic variant and outcome, preferably on an interpretable scale | No page | These details are presented in Supplementary Table 1. For instance, the phenotype "Back pain For 3+ months" indicates chronic pain lasting three or more months, localized in the anatomical region of the back. |
|  | b) | Report MR estimates of the relationship between exposure and outcome, and the measures of uncertainty from the MR analysis, on an interpretable scale, such as odds ratio or relative risk per SD difference | Supplementary table 6-9 | Detail SD difference and estimates could find in the Supplementary table 6-9. |
|  | c) | If relevant, consider translating estimates of relative risk into absolute risk for a meaningful time period | No Applicable |  |
|  | d) | Consider plots to visualize results (e.g. forest plot, scatterplot of associations between genetic variants and outcome versus between genetic variants and exposure) | 29, 30, 33 | Figure 3, 4, 7 |
| 12 | **Assessment of assumptions** |  |  |  |
|  | a) | Report the assessment of the validity of the assumptions | 13-14; Table 2;  Table S10 – S17 | MR PRESSO didn’t identify heterogeneity among the significant (Table 2) and potential microbiota (Supplementary table S10 - S13). Moreover, the Leave-one-out analysis revealed no significant differences in SNP effects (Fig.8). Furthermore, the MR Egger regression did not yield any evidence of horizontal pleiotropy (P > 0.05), and all F-statistical values exceeded 10 (Table 2, Supplementary table S10 - S13). The estimated values from the five methods (IVW < 0.05) can be found in Supplementary table S14 - S17. |
|  | b) | Report any additional statistics (e.g., assessments of heterogeneity across genetic variants, such as *I^2^*, Q statistic or E-value) | 13,14; Table 2; | Similarly, Cochran's Q test indicated the absence of heterogeneity across the studies (Table 2, Supplementary table S10 - S13)…… |
| 13 | **Sensitivity analyses and additional analyses** |  |  |  |
|  | a) | Report any sensitivity analyses to assess the robustness of the main results to violations of the assumptions | 13,14;  Table 2;  Table S10 - S17 | MR PRESSO didn’t identify heterogeneity among the significant (Table 2) and potential microbiota (Supplementary table S10 - S13). Furthermore, the MR Egger regression did not yield any evidence of horizontal pleiotropy (P > 0.05), and all F-statistical values exceeded 10 (Table 2, Supplementary table S10 - S13). The estimated values from the five methods (IVW < 0.05) can be found in Supplementary table S14 - S17. |
|  | b) | Report results from other sensitivity analyses or additional analyses | 14 | The estimated values from the five methods (IVW < 0.05) can be found in Supplementary table S14 - S17. |
|  | c) | Report any assessment of direction of causal relationship (e.g., bidirectional MR) | 10-13 | Casual effect of gut microbiome on chronic pain  Casual effect of chronic pain on gut microbiome  Potential causal effects between gut microbiota and CP |
|  | d) | When relevant, report and compare with estimates from non-MR analyses | No Applicable |  |
|  | e) | Consider additional plots to visualize results (e.g., leave-one-out analyses) | Figure 3,4,5,6, | Figure3,4,5,6, |
|  | **DISCUSSION** |  |  |  |
| 14 | **Key results** | Summarize key results with reference to study objectives | 14 | this is the first systematic study that utilizes a two-sample Mendelian randomization approach to assess the genetic associations between gut microbiota and site-specific CP. This research provides robust evidence of the role of genetics in predicting gut microbiota abundance in the development of site-specific CP. The existence of the microbiota-gut-brain axis theory offers a potential avenue for investigating the relationship between diseases and gut microbiota |
| 15 | **Limitations** | Discuss limitations of the study, taking into account the validity of the IV assumptions, other sources of potential bias, and imprecision. Discuss both direction and magnitude of any potential bias and any efforts to address them | 16 | We must acknowledge several limitations in our study……. |
| 16 | **Interpretation** |  |  |  |
|  | a) | Meaning: Give a cautious overall interpretation of results in the context of their limitations and in comparison with other studies | 14-15 | Genus *Methanobrevibacter* is considered a major contributor to methane production in the human gut. Individuals with high methane levels in their gut can have Genus *Methanobrevibacter* abundance up to 1000 times higher than normal individuals…….. |
|  | b) | Mechanism: Discuss underlying biological mechanisms that could drive a potential causal relationship between the investigated exposure and the outcome, and whether the gene-environment equivalence assumption is reasonable. Use causal language carefully, clarifying that IV estimates may provide causal effects only under certain assumptions | 14-15 | A recent study also showed a significant reduction in the abundance of *Genus Parabacteroides* in the gut of rheumatoid arthritis patients, and this decrease was positively correlated with disease activity. *Genus Parabacteroides* may alleviate rheumatoid arthritis by directly or indirectly affecting the differentiation of T helper 17 cells through secondary bile acids. Rheumatoid arthritis patients often experience CP |
|  | c) | Clinical relevance: Discuss whether the results have clinical or public policy relevance, and to what extent they inform effect sizes of possible interventions | 17 | Our discoveries strongly imply that there could be a direct correlation between distinct chronic regional pain and specific gut microbiota compositions. By modulating the biological abundance of corresponding microbiota, precise treatment goals may be achieved. |
| 17 | **Generalizability** | Discuss the generalizability of the study results (a) to other populations, (b) across other exposure periods/timings, and (c) across other levels of exposure | 16 | the GWAS data we primarily utilized in our study are derived from populations of European ancestry, including both gut microbiota and chronic pain data. Therefore, the generalizability of our findings to other ethnic groups should be approached with caution. |
|  | **OTHER INFORMATION** |  |  |  |
| 18 | **Funding** | Describe sources of funding and the role of funders in the present study and, if applicable, sources of funding for the databases and original study or studies on which the present study is based | 18 | This work was supported by grants from National Natural Science Foundation of China (No. 31772709) and Hubei Natural Science Foundation (2015CFB316) of China. |
| 19 | **Data and data sharing** | Provide the data used to perform all analyses or report where and how the data can be accessed, and reference these sources in the article. Provide the statistical code needed to reproduce the results in the article, or report whether the code is publicly accessible and if so, where | 17 | Data availability statement |
| 20 | **Conflicts of Interest** | All authors should declare all potential conflicts of interest |  | The authors declare that they have no competing interests. |

This checklist is copyrighted by the Equator Network under the Creative Commons Attribution 3.0 Unported (CC BY 3.0) license.

1. Skrivankova VW, Richmond RC, Woolf BAR, Yarmolinsky J, Davies NM, Swanson SA, et al. Strengthening the Reporting of Observational Studies in Epidemiology using Mendelian Randomization (STROBE-MR) Statement. JAMA. 2021; under review.

2. Skrivankova VW, Richmond RC, Woolf BAR, Davies NM, Swanson SA, VanderWeele TJ, et al. Strengthening the Reporting of Observational Studies in Epidemiology using Mendelian Randomisation (STROBE-MR): Explanation and Elaboration. BMJ. 2021;375: n2233.
